# Aged mice show an increased mortality after anesthesia with a standard dose of ketamine/xylazine

**DOI:** 10.1186/s42826-019-0008-y

**Published:** 2019-07-24

**Authors:** Sandra Schuetze, Anja Manig, Sandra Ribes, Roland Nau

**Affiliations:** 1 Institute of Neuropathology, University Medical Center Göttingen, Robert-Koch-Str. 40, D-37075 Göttingen, Germany; 2 Department of Geriatrics, AGAPLESION Frankfurter Diakonie Kliniken, 60431 Frankfurt am Main, Germany; 3 Department of Clinical Neurophysiology, University Medical Center Göttingen, 37075 Göttingen, Germany; 4 Department of Geriatrics, Evangelisches Krankenhaus Göttingen-Weende, 37075 Göttingen, Germany

**Keywords:** Aging, Anesthetics, Geriatric mouse model, Mortality, C57BL/6

## Abstract

Geriatric animal models are crucial for a better understanding and an improved therapy of age-related diseases. We observed a high mortality of aged mice after anesthesia with a standard dose of ketamine/xylazine, an anesthetic regimen frequently used in laboratory veterinary medicine.

C57BL/6-N mice at the age of 2.14 ± 0.23 months (young mice) and 26.31 ± 2.15 months (aged mice) were anesthetized by intraperitoneal injection of 2 mg ketamine and 0.2 mg xylazine. 4 of 26 aged mice (15.4%) but none of 26 young mice died within 15 min after injection of the anesthetics. The weight of aged mice was significantly higher than that of young mice (32.8 ± 5.4 g versus 23.2 ± 3.4 g, *p* < 0.0001). Thus, aged mice received lower doses of anesthetics in relation to their body weight which are within the lower range of doses recommended in the literature or even beneath. There were no differences between deceased and surviving aged mice concerning their sex, weight and their motor performance prior to anesthesia.

Our data clearly show an age-related increase of mortality upon anesthesia with low standard doses of ketamine/xylazine. Assessment of weight and motor performance did not help to predict vulnerability of aged mice to the anesthetics. Caution is necessary when this common anesthetic regimen is applied in aged mice: lower doses or the use of alternative anesthetics should be considered to avoid unexpected mortality.

The present data from our geriatric mouse model strongly corroborate an age-adjusted reduction of anesthetic doses to reduce anesthesia-related mortality in aged individuals.

## Introduction

The increased life expectancy is going hand in hand with an increase of age-related diseases, such as neurodegenerative diseases, cancer, and atherosclerosis [1]. Aged persons are more vulnerable to infections and other external stressors [2, 3]. Because of the age-related decline of organ functions and age-related changes of pharmacokinetic and pharmacodynamic features, elderly individuals react differently to therapeutics and anesthetics [4, 5]. To improve therapies for the growing group of geriatric persons, age-related diseases and conditions require proper scientific investigation. In clinical studies, nowadays there is a trend to pay more attention to persons at an age over 80 years, which have been excluded from many studies in the past [6, 7]. Geriatric animal models are needed for a better understanding of age-related changes and processes. Recently, geriatric mouse models for frailty and sarcopenia including C57BL/6 mice up to an age of 28 months have been established [8–10]. Thus, aged mice become increasingly important for basic research. This is reflected by the fact that animal breeding companies started to offer aged mice up to an age of more than 24 months. Many interventions during mouse experiments require anesthesia, and the ketamine/xylazine regimen is widely used [11–14]. There are some studies which primarily focus on the effects of these anesthetics on the mouse creature [15–18], the influence of mouse strain and sex on the susceptibility to anesthetics [19], or the optimization of anesthesia protocols in mice [20–22]. Although advanced age over 18 months has been considered to influence susceptibility of mice to anesthetics [13], to our knowledge, experimental studies or recommendations primarily addressing anesthetic regimes for geriatric mice do not exist so far. ARRIVE guidelines specify to pay attention to anesthesia [23]. However, the crucial role of anesthetics is often ignored or underestimated in the experimental design and later publication of animal research models [24, 25].

We established a geriatric mouse model for *Escherichia coli (E. coli)* meningitis [26], in which young and aged mice received intraperitoneal anesthesia with standard doses of ketamine/xylazine before intracerebral injection of *E. coli* K1. During these experiments, we observed a substantial mortality of aged mice under this standard anesthetic regimen frequently used in laboratory veterinary medicine. We consider this observation highly relevant for our further work with geriatric mouse models and for other researchers performing experiments with aged mice.

## Methods/ experimental

### Animals

Animal experiments were approved by the Animal Care Committee of the University Medical Center Göttingen, Germany, and by the Niedersächsisches Landesamt für Verbraucherschutz und Lebensmittelsicherheit (LAVES), Braunschweig, Lower Saxony, Germany.

C57BL/6-N mice were bred in the animal care facility (Zentrale Tierexperimentelle Einrichtung) of the of the University Medical Center Göttingen, Germany, and housed under a 12:12 h light:dark cyle, 20 °C room temperature and 55% humidity in compatible groups of maximum 5 animals until they reached the intended age. They were provided with free choice standard rodent diet and bottled tap water. 26 C57BL/6-N mice at the age of 2.14 ± 0.23 months (young mice) and 26 C57BL/6-N mice at the age of 26.31 ± 2.15 months (aged mice) received anesthesia. Prior to anesthesia, all mice were weighed and their motor performance was assessed using the tight rope test.

### Tight rope test

Mice were placed with their front paws in the middle of a 60 cm long rope tightly spanned about 60 cm above a padded floor. The time until one end of the rope was reached was measured, and a performance score was assigned (minimum score 0, maximum score 20). Mice reaching one end in ≤6 s were given score 0. An additional point was added for every 6 additional seconds needed. Mice hanging for 60 s on the rope but not reaching one end were given a score of 10. Mice which fell down before that time received additional points to the 10 points for every 6 s falling earlier than 60 s [27].

### Anesthetic dilution and administration

Ketamine 10% (100 mg/ml; Medistar, Ascheberg, Germany) and xylazine 2% (20 mg/ml; Riemser, Greifswald, Germany) were combined in a single insulin syringe (2 parts of ketamine and 1 part of xylazine). Mice were manually restrained, and 30 μl of the anesthetic mixture were injected intraperitoneally into the right lower quadrant of the abdomen using a 25-gauge needle. Independently of the body weight, each mouse received 2 mg ketamine and 0.2 mg xylazine.

### Statistics

GraphPad Prism 5.0 Software (GraphPad Software, San Diego, California, USA) was used to perform statistical analyses and graphical presentation. Log-rank test was performed for the comparison of survival curves. Weights of mice and doses of anesthetics are expressed as means±standard deviations (SD) and were compared using the Student’s *t*-test. The tight rope test scores are expressed as medians (25th percentile/75th percentile). Mann-Whitney *U*-test was performed to compare the tight rope test scores between the groups. Sex distributions were compared using the Chi-squared test. *P*-values < 0.05 were considered statistically significant.

## Results

**Fig. 1:**
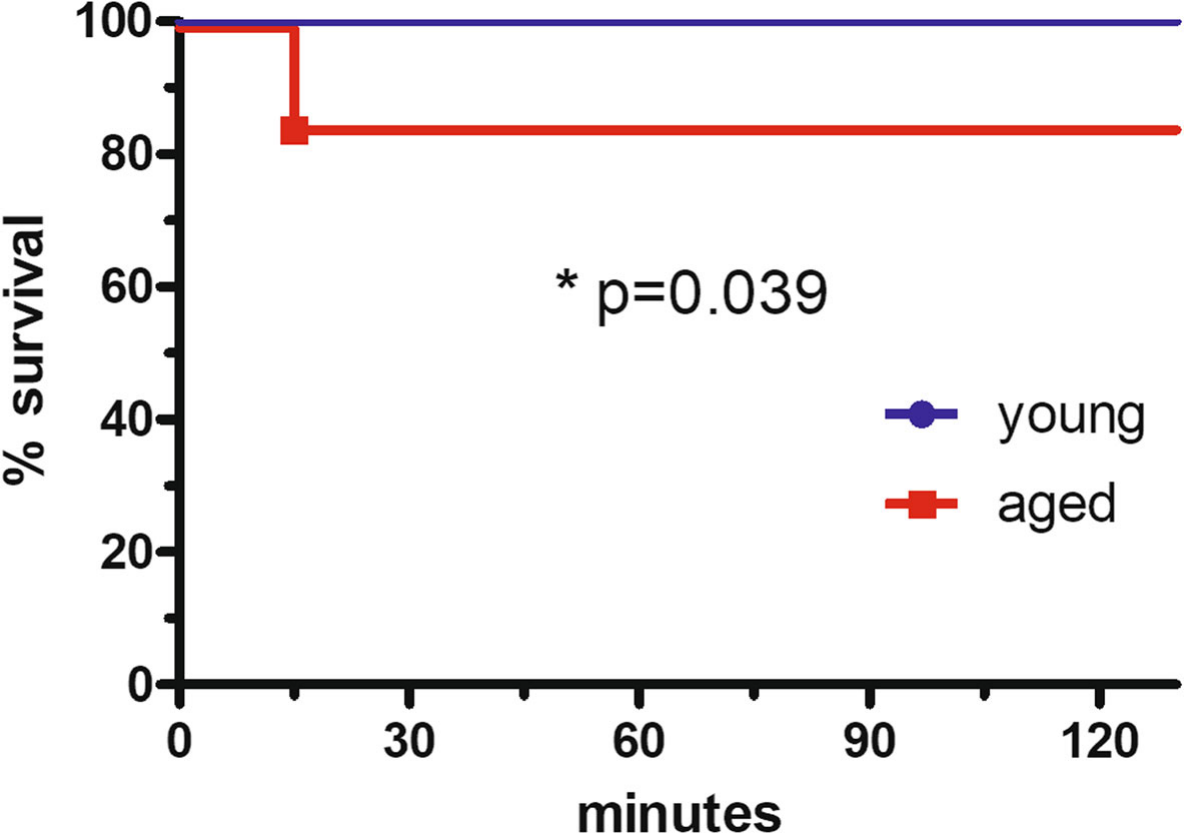
Mortality of young and aged mice after anesthesia with ketamine/xylazine. Kaplan-Meier curves of young mice (2 months) and aged mice (26 months) after intraperitoneal injection of 2 mg ketamine and 0.2 mg xylazine. 15.4% of the aged mice but none of the young mice died after anesthesia (log-rank test: *p* = 0.039)

4 of 26 aged mice (15.4%) died within 15 min after injection of ketamine/xylazine (2 mg/0.2 mg), whereas none of the 26 young mice died from the anesthesia (*p* = 0.039; Fig. 1).

**Fig. 2:**
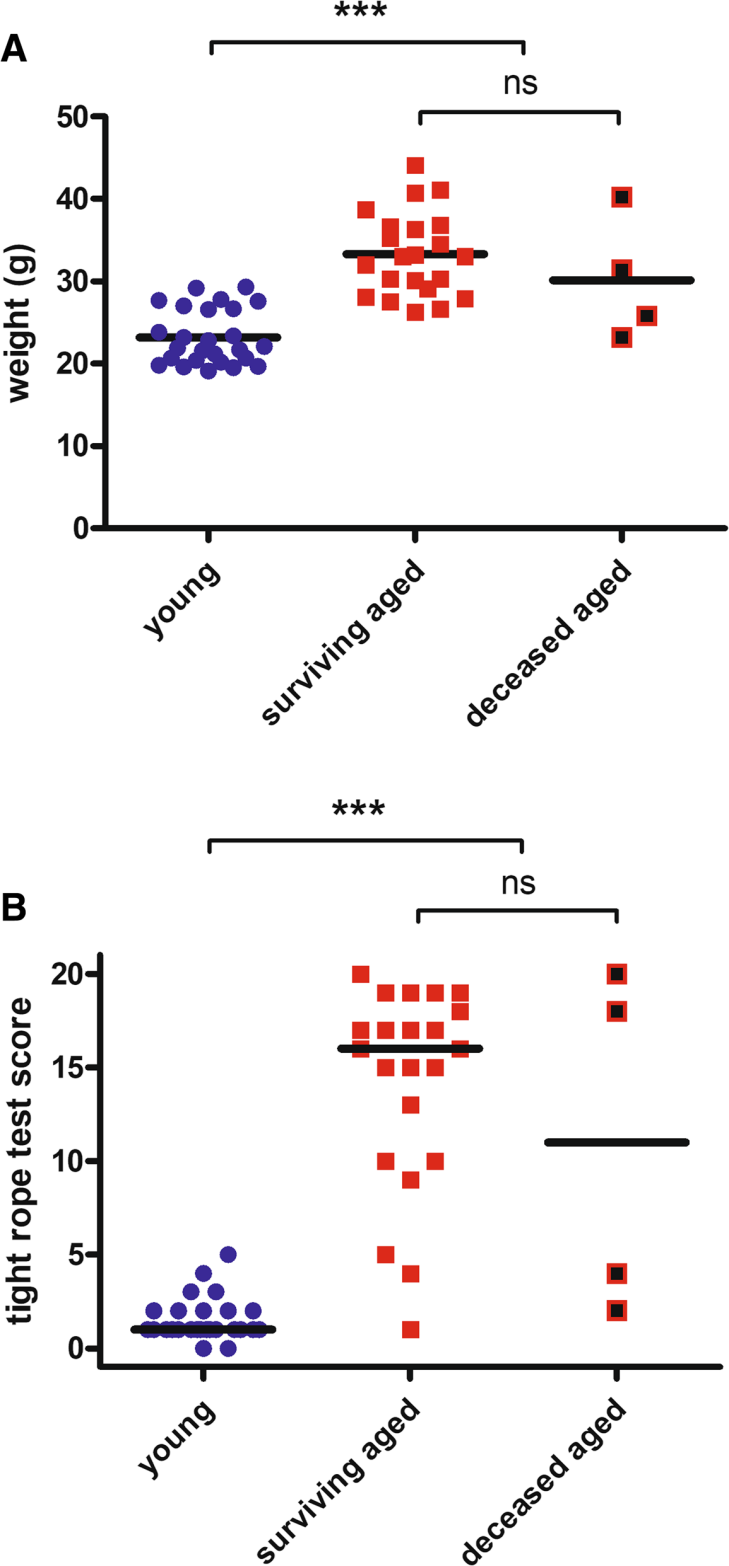
Weight and motor performance. **a**: Weight of aged mice was significantly higher than that of young mice (32.8 ± 5.4 g versus 23.2 ± 3.4 g; *p* < 0.0001). Weight of surviving and deceased aged mice did not differ (33.3 ± 5.0 g versus 30.2 ± 7.5 g; *p* = 0.30). Data are presented as individual values and means and were compared using Student’s *t*-test (***p < 0.0001; ns = not significant). **b**: Tight rope test scores [medians (25th percentile/75th percentile)] of aged mice were significantly higher than those of young mice [16(9.75/18.25) versus 1(1/2); *p* < 0.0001]. Tight rope test scores of surviving and deceased aged mice did not differ [16(10/18.25) versus 11(2.5/19.5); *p* = 0.83]. Data are presented as individual values and medians and were compared using the Mann-Whitney *U*-test (***p < 0.0001; ns = not significant)

Sex distribution was similar in both groups [young mice: 17 of 26 mice female (65%), aged mice: 18 of 26 mice female (69%); Table 1]. Weight of aged mice (32.8 ± 5.4 g) was substantially higher than that of young mice (23.2 ± 3.4 g; *p* < 0.0001; Fig. 2a, Table 1). Young mice showed a better motor performance than aged mice as assessed by the tight rope test, with lower scores indicating a better performance: tight rope test scores [medians (25th percentile/75th percentile)] of young mice were significantly lower than those of aged mice [1(1/2) versus 16(9.75/18.25); *p* < 0.0001; Fig. 2b]. There was no significant difference between surviving and deceased aged mice concerning their body weight (33.3 ± 5.0 g versus 30.2 ± 7.5 g; *p* = 0.30; Fig. 2a, Table 1). and their tight rope test scores [16(10/18.25) versus 11(2.5/19.5); *p* = 0.83; Fig. 2b].

**Table 1:** Comparison of age, sex distribution, and doses of anesthetics between young and aged mice (left column) and between surviving and deceased aged mice (right column)

	young (*n* = 26)	aged (*n* = 26)	*p*	surviving aged (*n* = 22)	deceased aged (*n* = 4)	*p*
age in months (mean ± SD)	2.14 ± 0.23	26.31 ± 2.15	*< 0.0001*	26.18 ± 2.26	27.0 ± 1.35	*0.49*
sex distribution [female: n (%)]	17 (65%)	18 (69%)	*0.77*	15 (68%)	3 (75%)	*0.79*
weight in g (mean ± SD)	23.2 ± 3.4	32.8 ± 5.4	*< 0.0001*	33.3 ± 5.0	30.2 ± 7.5	*0.30*
ketamine dose (mg/kg)	87.9 ± 12.0	62.6 ± 10.3	*< 0.0001*	61.4 ± 9.0	69.3 ± 16.0	*0.17*
xylazine dose (mg/kg)	8.8 ± 1.2	6.3 ± 1.0	*< 0.0001*	6.2 ± 0.9	6.9 ± 1.6	*0.17*

Ages of surviving and deceased aged mice did not differ significantly (*p* = 0.49; Table 1), and sex distribution of surviving and deceased aged mice was similar (*p* = 0.79; Table 1).

In relation to their body weight, aged mice received approximately 70% of the doses of ketamine and xylazine administered to young mice (aged mice: 62.6 ± 10.3 mg/kg ketamine, 6.3 ± 1.0 mg/kg xylazine; young mice: 87.9 ± 12.0 mg/kg ketamine, 8.8 ± 1.2 mg/kg xylazine; *p* < 0.0001; Table 1). Doses of the anesthetics did not differ significantly between surviving aged mice (61.4 ± 9.0 mg/kg ketamine, 6.2 ± 0.9 mg/kg xylazine) and deceased aged mice (69.3 ± 16.0 mg/kg ketamine, 6.9 ± 1.6 mg/kg xylazine; *p* = 0.17; Table 1).

## Discussion

Our data clearly show that a standard dose of ketamine/xylazine with no related severe adverse effects in young mice can be detrimental in aged animals. We observed a mortality of 15% in mice with an age of approximately 2 years, although aged mice received substantially lower doses of the anesthetics per body weight. Weight and motor performance assessed before anesthesia were not able to predict outcome.

The combination of ketamine and xylazine is one of the most frequently used anesthetic regimens in rodents [11–14]. Hypotension and heart rate decrease are the major adverse reactions of ketamine/xylazine in mice, whereas respiratory functions are less affected [14]. Widely used doses of ketamine 100 mg/kg and xylazine 10 mg/kg have been shown to result in a sufficient anesthetic depth and low respiratory depression [14]. These doses have also been used for anesthesia in our mouse meningitis models since many years without causing problems concerning mortality [27, 28]. Reported doses for ketamine/xylazine in mice range from 60 to 200 mg/kg ketamine and 4–20 mg/kg xylazine [11, 12]. In the present study, we induced anesthesia by injection of 2 mg ketamine and 0.2 mg xylazine per mouse independently of the body weight. Thus, the mouse with the lowest weight (19 g) received approximately 100/10 mg/kg ketamine/xylazine; all other mice received lower doses. The mouse with the highest weight (44 g) received only 45/4.5 mg/kg ketamine/xylazine. According to their higher body weight, aged mice received significantly lower doses than young mice (only approximately 70% of the doses of young mice) which are within the lower range of reported doses or even beneath [11, 12]. Although administered doses of anesthetics were not significantly different between surviving and deceased aged mice, in tendency, deceased mice received higher doses (69.3 ± 16.0/6.9 ± 1.6 mg/kg versus 61.4 ± 9.0/6.2 ± 0.9 mg/kg). All surviving mice developed sufficient depth of anesthesia to perform a painless intracerebral injection and recovered from anesthesia without apparent deficits. Injection of weight-adjusted amounts of ketamine and xylazine aiming at equal doses in young and aged animals (e.g. 100/10 mg/kg) might have resulted in a much higher mortality within the group of aged mice.

The avoidance of non-intended death of laboratory animals has highest priority for all researchers performing animal experiments. Therefore, an unintended mortality of 15% upon anesthesia in aged mice is not acceptable and must urgently be avoided in future experiments. It is not clear from our experiments, which parameters caused the increased vulnerability of aged mice to the anesthetics used. Easily to perform assessment of weight and motor function did not help to predict vulnerability of mice to ketamine and xylazine.

The NMDA antagonist ketamine leads to an increased heart rate and hypertension, the alpha-two adrenoceptor agonist xylazine has biphasic cardiovascular effects with bradycardia and hypertension within the first 15 min followed by hypotension and reduced cardiac excitability as well as arrhythmias [5]. The finding that death occurred within 15 min after injection of anesthetics allows excluding hypotension as cause of decease. Reduction of respiratory frequency and blood oxygen saturation was similar in young and aged rats after injection of ketamine/xylazine 80/10 mg/kg, whereas cardiac frequency was significantly more reduced in aged compared to young rats already shortly after injection of the anesthetics [29]. This suggests that most probably the early cardiac effects of the alpha-two agonist xylazine resulting in a more pronounced bradycardia in aged individuals led to death in aged mice.

Caution is necessary when this common anesthetic regimen is applied in aged mice. Weight-adjustment of anesthetics which is recommended in many anesthetic protocols should be avoided in geriatric mouse models as it most probably results in overdosing and dramatically unexpected high mortality of aged mice. However, age-corrected reduction of anesthetic doses seems to be essential to save the life of valuable aged mice. Our results suggest that only doses of ketamine/xylazine below 60/6 mg/kg should be used for anesthesia of aged mice. Furthermore, alternative anesthetic regimens should be evaluated in geriatric mice, e.g. regimens avoiding or reducing alpha-two agonists, such as xylazine, in order to minimize the unintended reduction of cardiac frequency which has been shown to be more pronounced in aged individuals [5, 29]. Isoflurane is recommended in many protocols, including anesthesia for brain interventions, as it allows a tight control of the anesthesia plane and a fast recovery after surgery [30]. However, isoflurane affects the expression of key neuroimmunomodulators in the hippocampus of aged mice and might contribute to the development of post-anesthesia cognitive impairment; furthermore, it leads to an impaired systemic immune response, e.g. by inhibition of macrophage functions and bacterial clearance [31]. Thus, isoflurane cannot be recommended for anesthesia in aged individuals, and particularly not for experiments addressing immunologic questions. The use of much lower doses of ketamine/xylazine (e.g. 10/1 mg/kg) followed by subcutaneous injection of the analgesic buprenorphine [32] and if necessary re-injection with ketamine alone [30] could be an alternative anesthetic regimen in order to avoid the potentially detrimental cardiovascular effects of xylazine.

Elderly patients are undergoing surgical procedures with increasing frequency [33, 34]. They are at a relatively higher risk of perioperative mortality and morbidity compared to younger patients [4, 35]. To what extent anesthesia is causative for this, is difficult to study in humans. Guidelines recommend a 25–50% reduction of anesthetics in elderly patients [34]. However, suggested age-correction of anesthetic doses often is not sufficiently performed in clinical practice, and this failure might contribute to increased perioperative morbidity and mortality [36, 37].

## Conclusions

The present data from our geriatric mouse model strongly corroborate the necessity of reduction of anesthetic doses and adaption of anesthetic regimens in aged individuals. Animal experiments specifically designed to compare effects of anesthetics in aged and young animals might help to identify parameters accounting for the increased susceptibility to anesthesia and to reduce the anesthesia-related perioperative mortality of aged individuals.

## Data Availability

The datasets used and/or analyzed during the current study are available from the corresponding author on reasonable request.

## Abbreviations

*E. coli*: *Escherichia coli*
SD: Standard deviation
